# Effect of Diet Supplementation with Two Yeast Cultures on Rumen Fermentation Parameters and Microbiota of Fattening Sheep In Vitro

**DOI:** 10.3390/microorganisms13030550

**Published:** 2025-02-28

**Authors:** Gulinizier Nueraihemaiti, Xiangdong Huo, Huiying Zhang, Honglin Shi, Yan Gao, Jun Zeng, Qing Lin, Kai Lou

**Affiliations:** 1 College of Life Sciences and Technology, Xinjiang University, Urumqi 830049, China; 17397642030@163.com; 2 Microbiology Institute of Microbiology, Xinjiang Academy of Agricultural Sciences, Urumqi 830091, China; huoxd@xaas.ac.cn (X.H.); 19993686824@163.com (H.Z.); 15509947314@163.com (H.S.); gaoyan19790826@outlook.com (Y.G.); leo924.student@sina.com (J.Z.); 3 Xinjiang Laboratory of Special Enviromental Microbiology, Urumqi 830091, China

**Keywords:** yeast culture, ruminal fermentation, fattening sheep, microbial community, volatile fatty acids

## Abstract

Yeast culture can improve ruminant health and reduce economic losses in intensive farming, but as a non-standardized product in China, its quality and efficacy vary significantly. In this study, a self-developed yeast culture was compared with a commercially available product using in vitro rumen fermentation and amplicon-based high-throughput sequencing to evaluate its effects on rumen fermentation parameters, microbial diversities, and community compositions in Hu sheep. The aim was to validate the efficacy and mechanisms of the self-developed yeast culture, produced with simplified raw materials and processes, on rumen function. The experiment was divided into four groups. In each 60 mL fermentation solution, the following treatments were added: 0.00 g high-concentrate diet (CK1 group, blank control), 0.40 g high-concentrate diet (CK2 group, basal diet control), 0.40 g high-concentrate diet supplemented with 5% XP yeast culture (XP group), and 0.40 g high-concentrate diet supplemented with 5% YC yeast culture (YC group). Gas production was measured every 4 h during fermentation. At the end of fermentation, pH, ammonia nitrogen, microbial protein, volatile fatty acids, and ruminal microbiota were determined. The results demonstrated the following. Compared to the CK2 group, both the XP and YC groups exhibited a significant increase (*p* < 0.05) in cumulative gas production and microbial protein content, while a significant decrease (*p* < 0.05) was observed in acetic acid content and the acetate-to-propionate ratio. The microbial protein content in the YC group was significantly higher (*p* < 0.05) than that in the XP group. Additionally, the content of valeric acid and isobutyric acid in the XP group was significantly higher (*p* < 0.05) compared to the other groups. The microbial community sequencing results revealed that the addition of yeast culture did not affect the alpha diversity index of rumen bacteria (*p* > 0.05); however, the addition of XP significantly reduced (*p* < 0.05) the richness of rumen fungal communities. At the phylum and genus levels, the relative abundance of multiple functional bacteria improved after adding YC. In summary, under the conditions of in vitro rumen fermentation with high-concentrate diets, adding 5% XP and YC yeast cultures both promoted rumen fermentation. The rumen fermentation type changed from the acetic acid type to the propionic acid type, which regulated rumen microbial composition and thereby improved dietary digestion efficiency. Notably, YC significantly increased the relative abundance of functional microbial communities compared to XP. These findings provide a theoretical and practical foundation for optimizing the large-scale breeding of Hu sheep.

## 1. Introduction

China is the largest producer and consumer of mutton sheep [1]. According to official statistics, in 2023, the number of sheep slaughtered in China was 338.64 million, and the domestic mutton production reached 5.31 million tons [2]. However, an additional 434,000 tons of mutton were imported to meet the demands of the consumer market [3]. The substantial production and consumption demands, coupled with the advantages of intensive housing systems in terms of efficiency and environmental sustainability, have made the replacement of traditional grazing a growing trend [4]. Due to geographical and climatic constraints, China faces a shortage of high-quality forage resources [5]. To maximize production performance and economic benefits, feeding high-concentrate diets with higher energy density has become a key strategy in intensive ruminant farming [6]. However, long-term feeding of high-concentrate diets leads to the rapid fermentation of large amounts of readily fermentable carbohydrates in the rumen, causing the accumulation of volatile fatty acids (VFAs) and lactic acid [7]. This results in a decline in rumen pH below normal levels, leading to metabolic acidosis and even triggering more severe secondary diseases [8]. It is a common cause of morbidity and mortality in both small and large ruminant populations [8]. Since ruminal acidosis is a continuum of disorders with varying severity, it may not exhibit obvious clinical signs in the early stages, making it difficult to detect within a short period [5]. It severely impacts the production performance and health status of ruminants, posing major challenges to the sustainable development of the livestock industry. Therefore, preventing ruminal acidosis is crucial for ensuring animal welfare and production. At present, buffering agents, lactic acid bacteria, and yeast products can be used to prevent ruminal acidosis [6,9]. Yeast products have gained significant attention as a safe and healthy feed additive in animal production and nutritional health.

Previous studies have found that yeast culture (YC) can mitigate the adverse effects of acidosis in ruminants, potentially improving animal growth performance and health [6]. However, due to product differences, yeast cultures exhibit significant variations in their effects on different animals. For example, some yeast cultures can increase the ruminal pH of sheep with acute acidosis, reduce ruminal osmotic pressure, and improve acute acidosis [10]. Some yeast cultures enhance animal production performance by optimizing the structure of rumen microbiota. For example, adding yeast cultures to the diet of dairy cows can increase the relative abundance of protein-synthesizing bacteria and cellulose-degrading bacteria in the rumen, thus promoting the utilization of nitrogen-containing substances in feed [11,12]. Some yeast cultures have no significant effect on the growth performance of fattening cattle and sheep, but they can enhance antioxidant capacity and increase the digestibility of crude protein and neutral detergent fiber [13,14]. These differences may arise from factors, such as yeast species, yeast culture production processes, and feeding management practices, such as diet composition and yeast culture additive dosage. Yeast culture is a non-standardized product in China, and there are currently no relevant national standards issued in the country [15]. As a result, the quality and efficacy of market products exhibit considerable variability. In the early stages of the project, a yeast culture with simplified fermentation of raw materials and processes was developed. Non-targeted metabolomics analysis has identified that it contains multiple metabolic components with pharmacological and physiological effects, indicating potential application value. However, its effects on ruminants have not yet been validated.

Hu sheep is a unique sheep breed in China, known for its high reproductive performance, fast growth rate, and strong environmental adaptability, making it one of the mainstream meat sheep breeds for large-scale farming [16]. Research indicates that feeding mutton sheep with high-concentrate diets results in a reduction in the abundance and diversity of rumen microorganisms, as well as metabolic disorders [17]. In this study, we compared a self-developed yeast culture with a commercially available product, hypothesizing that both yeast cultures could improve rumen fermentation and mitigate the reduction in microbial diversity under high-concentrate feeding conditions. To test this hypothesis, in vitro rumen fermentation and amplicon-based high-throughput sequencing were employed to evaluate the effects and mechanisms of the self-developed yeast culture, produced with simplified raw materials and processes, on the rumen function of Hu sheep.

## 2. Materials and Methods

### 2.1. Ethical Statement Experimental

This study has been approved by the Animal Ethics Committee of the Institute of Animal Husbandry, Xinjiang Academy of Animal Sciences (Approval Number: Dong Lun [2024] No. 09).

### 2.2. Design and In Vitro Rumen Fermentation

The yeast culture product XP used in this study was purchased from Diamond V Biological Fermentation Engineering Technology (Shenzhen) Co., Ltd. (Shenzhen, China). The self-developed yeast culture YC was fermented by *Saccharomyces cerevisiae* in the early stage of our laboratory. The high-concentrate diets were composed of 45% total mixed ration, 45% corn flour, and 10% grass powder. The high-concentrate diets with this formulation have a starch level of 42%, a neutral detergent fiber (NDF) of 29.35%, an acid detergent fiber (ADF) of 14.60%, and a metabolizable energy (ME) of 10.67 MJ/kg. The total mixed ration was prepared according to China’s “Feeding Standard of Meat-producing Sheep and Goats (NY/T 816-2021)” [18] (Table 1).

In the in vitro fermentation experiment, three anaerobic fermentation bottles sealed with rubber stoppers and connected by glass conduits and rubber tubes were used to simulate rumen fermentation in vitro. The fermentation solution, along with the added diet and yeast culture, underwent anaerobic fermentation in the first glass bottle. The second glass bottle was filled with water, while the third glass bottle collected and measured gas production using the water displacement method.

The preparation of the in vitro fermentation solution was as follows. The artificial rumen nutrient solution was prepared according to the method of Menke et al. [19]. CO_2_ was passed until the color of the nutrient solution became colorless and transparent, and it was preheated in a 39 °C water bath for later use. Five healthy fattening Hu sheep of similar body conditions were selected as ruminal fluid donors. The diet composition for donor sheep was the same as that in the in vitro fermentation experiment. All donor animals were managed under the same feeding conditions. Rumen fluid was collected via stomach tube aspiration prior to morning feeding on the day of the experiment. The rumen fluid was filtered through four layers of gauze, transferred into a thermos cup filled with CO_2_ at 39 °C, and quickly returned to the laboratory. The in vitro fermentation solution was prepared by mixing the artificial rumen nutrient solution and rumen fluid in a 2:1 ratio, and CO_2_ was continuously injected to maintain an anaerobic environment. Four experimental groups were established. Control groups included 0.00 g high-concentrate diets (CK1 group, blank control) and 0.40 g high-concentrate diets (CK2 group, basal diet control). Trial groups included 0.40 g high-concentrate diets + 5% XP yeast culture (XP group) and 0.40 g high-concentrate diets+ 5% YC yeast culture (YC group). Each treatment group was added to 60 mL in vitro fermentation solution. After the bottle was filled with CO_2_, it was quickly sealed. The glass bottles were incubated in a constant temperature shaker at 39 °C for 48 h at 30 r/min, and each group was repeated 3 times.

### 2.3. Collection of Fermentation Broth Samples and Determination of Rumen Fermentation Parameters

During the incubation process, gas production was recorded at 4 h intervals (0, 4, 8, 12, 16, 20, 24, 28, 32, 36, 40, 44, and 48 h) using the water displacement method. After 48 h, the glass bottles were removed, and the fermentation was terminated in an ice water bath. The pH value was immediately measured. The fermentation solution was collected into 10 mL sterile centrifuge tubes and stored at −80 °C for subsequent analysis of ammonia nitrogen, microbial protein, volatile fatty acids, and ruminal microbiota. The pH value of the in vitro fermentation solution was measured by a pHS-3C desktop digital display acidity meter. The concentration of ammonia nitrogen was determined using the colorimetric method, and the absorbance was measured at a wavelength of 700 nm using a spectrophotometer [20]. The ammonia nitrogen concentration was calculated based on a standard curve. The content of microbial protein was determined by Bradford’s Coomassie Brilliant Blue method, the absorbance was determined by a spectrophotometer at 595 nm, and the microbial protein concentration was calculated according to the standard curve of bovine serum albumin (BSA) [21]. According to the method described by Wang Wenji [22], the fermentation solution was pretreated, and the VFA content in the fermentation solution was determined using a Baiqu GC-2030 gas chromatograph (Kyoto, Japan).

### 2.4. Microbiota Community Analysis

In vitro fermentation solution samples stored at −80 °C were sent to Beijing Novogene Bioinformatics Technology Co., Ltd. (Beijing, China) for DNA extraction, amplification, and sequencing. The specific steps were as follows. Total genomic DNA from in vitro fermentation solution samples was extracted by the CTAB method. The concentration and purity of the extracted DNA were assessed by electrophoresis on 1% agarose gel, and an appropriate amount of qualified DNA sample was then diluted to 1 ng/µL. Diluted genomic DNA was used as a template, and the bacterial primers 341F (5′-CCTAYGGGGRBGCASCAG-3′) and 806R (5′-GGACTACNNGGGGTATCTAAT-3′) were used to PCR amplify the V3-V4 region. In addition, the fungal primers ITS5-1737F (5′-GGAAGTAAAAGTCGTAACAAGG-3′) and ITS2-2043R (5′-GCTGCGTTCTTCATCGATGC-3′) were utilized to PCR amplify the ITS1-5F region [23]. Amplified PCR products were detected using 2% agarose gel electrophoresis, and the qualified PCR products were purified by magnetic beads. The equal amounts were mixed according to the PCR product concentration, after fully mixed, the PCR products were detected and the target bands recovered. PE250 was sequenced by the NovaSeq6000 platform (Illumina, San Diego, CA, USA).

The effective sequences obtained by filtering low-quality and chimeric sequences were denoised using the DADA2 module in QIIME2 software (version QIIME2-202202) to obtain the final ASVs (Amplicon Sequence Variants), and representative sequences of these ASVs were subjected to species annotation [24,25]. The Silva (138.1) database was used for bacterial annotation, and the Unite (v9.0) database was used for fungal annotation [26,27]. QIIME2 software was used to calculate the observed feature, Shannon index, Simpson index, Good’s coverage, and Chao1 index. The β diversity was evaluated using QIIME2 software and based on the unweighted Unifrac distance. In addition, one-way ANOVA was used to compare the differences in the relative abundance and diversity of microorganisms, and linear discriminant analysis effect size (LEfSe) and *t*-tests were applied to evaluate differentially abundant microorganisms.

### 2.5. Statistical Analysis

Before analysis, all data were tested for normality and homogeneity of variance. All data were processed using Excel 2016 and statistically analyzed using SPSS 23.0 software. One-way ANOVA was used to compare data, and Duncan’s multiple comparison was used to assess significant differences between groups. The results were presented as mean ± standard deviation, with *p* < 0.05 indicating significant differences. In order to analyze the differences in microbial community structure, PERMANOVA was used to evaluate the significance of differences between groups. The generation of Venn diagrams was performed using the VennDiagram function in R. The relative abundance distribution stacked bar plots of the top 10 species at the phylum and genus levels for each group were plotted using the SVG module in Perl. Principal Coordinate Analysis (PCoA) was computed using the ade4 package and visualized with the ggplot2 package in R software (v4.0.3). Differentially abundant rumen microbiota across groups were identified using linear discriminant analysis effect size (LEfSe) and *t*-tests. LEfSe analysis was performed and visualized with LEfSe software (2.2), applying a significance threshold of LDA score (LDA) ≥ 4 and *p* < 0.05.

## 3. Results

### 3.1. In Vitro Fermentation Parameters

**Figure 1:**
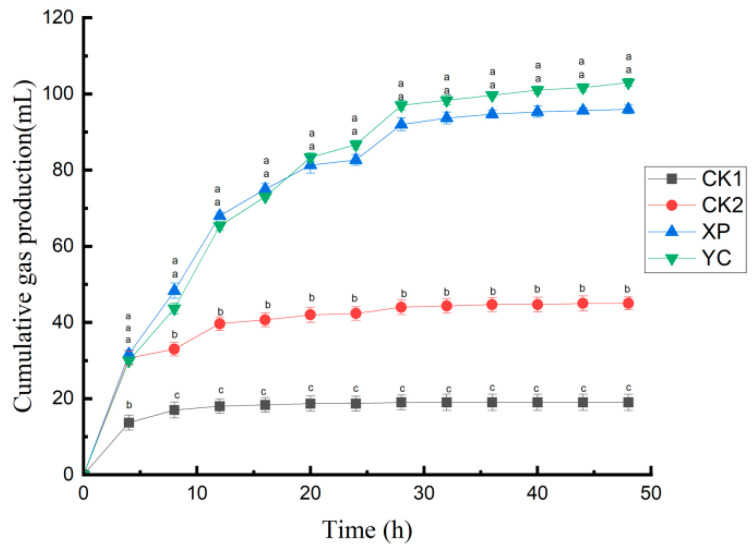
Cumulative gas production during 48 h in vitro fermentation. At each timepoint, letters a, b, and c describe significant differences at *p* < 0.05.

The ruminal fermentation parameters of the CK1 group were significantly lower than those of other groups (*p* < 0.05), except for pH and isovaleric acid content, after 48 h in vitro fermentation (Table 2). Compared with the CK2 group, adding yeast culture significantly increased (*p* < 0.05) cumulative gas production and microbial protein content, while significantly reducing (*p* < 0.05) acetic acid content and the acetate-to-propionate ratio. The concentrations of isobutyric acid and valeric acid in the XP group were significantly higher (*p* < 0.05) than those in other groups. The ruminal pH and microbial protein content in the YC group were significantly increased (*p* < 0.05) compared with the XP group. As shown in Figure 1, cumulative gas production in each group exhibited an increasing trend during the initial phase of 0 to 4 h. At 8 h, the cumulative gas production in the XP and YC groups was significantly higher (*p* < 0.05) than that in the CK1 and CK2 groups and continued to rise. The upward trend of cumulative gas production in the CK2 group at 12 h slowed down, while that in the XP group and the YC group at 28 h slowed down.

### 3.2. Rumen Bacterial Diversity Analysis

**Figure 2:**
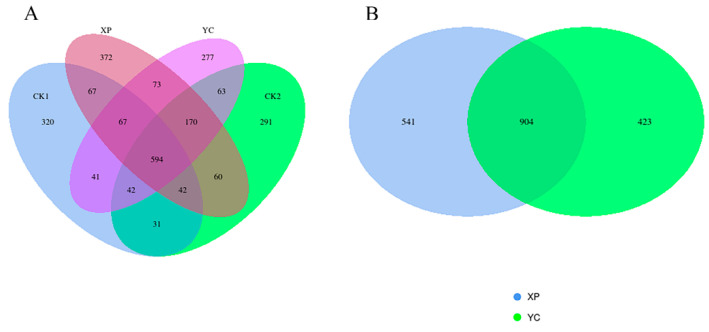
Venn diagram for all groups (**A**) and Venn diagram for trial groups (**B**) of ruminal bacteria.

A total of 2510 ASVs were detected in the fermentation solution of the four groups, with 1204, 1293, 1445, and 1327 ASVs in the CK1, CK2, XP, and YC groups, respectively (Figure 2). The shared ASVs were 594, accounting for 24% of the total ASVs, and the unique ASVs were 320, 291, 372, and 277, accounting for 27.00%, 23.00%, 25%, 70%, and 20.90% of the total number of ASVs, respectively. Between the XP and YC trial groups, there were 904 shared ASVs, accounting for 48.40% of the total ASVs in the trial groups. The above results indicated that adding yeast culture altered the ASV composition of the bacterial community in the fermentation solution.

**Figure 3:**
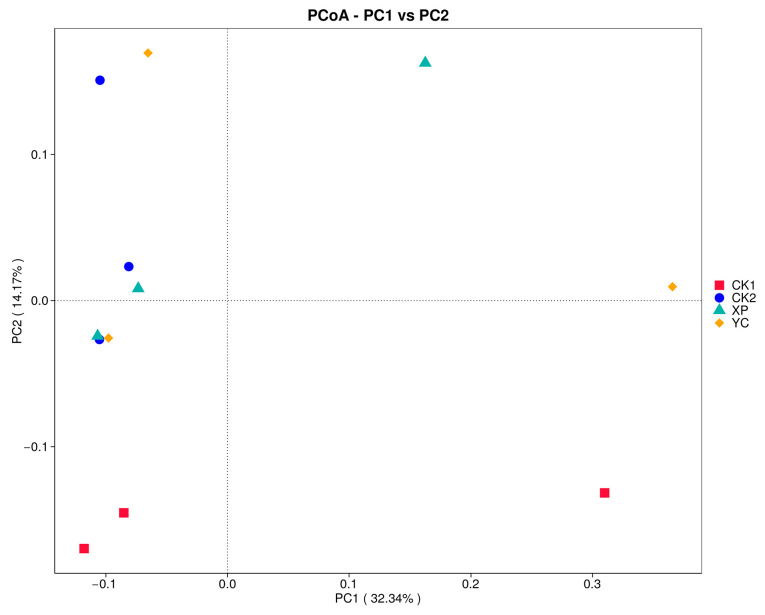
PCoA analysis of rumen bacteria.

The influences of two yeast cultures on the alpha and beta diversity of bacterial communities in ruminal fermentation solutions are shown in Table 3 and Figure 3, respectively. The results showed that Good’s coverage of the four groups was all equal to 1, which can accurately reflect the composition of most species in the rumen of Hu sheep. The Shannon index and Simpson index of the CK1 group were significantly lower (*p* < 0.05) than those of other groups. There were no significant differences in the alpha diversity indexes among the CK2, XP, and YC groups (*p* > 0.05), but the values in the XP and YC groups were higher than those in the control groups (Table 3). The PCoA results indicated that the coordinate position of the yeast culture treatments made changes, suggesting that adding yeast culture to the diets of mutton sheep had an impact on rumen microbiota composition (Figure 3). PERMNOVA further demonstrated that there were significant differences (*p* < 0.05) in the bacterial profiles among the four groups (*p* = 0.034).

**Figure 4:**
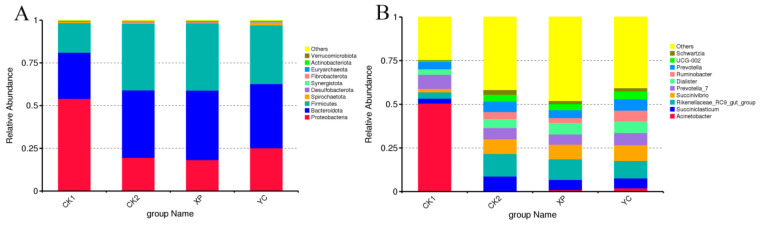
Bar chart of the relative abundance of species at the phylum (**A**) and genus (**B**) levels of rumen bacteria.

Through ASV annotation and comparison, a total of 19 phyla and 190 genera were detected in 12 samples from the four groups (Figure 4). Proteobacteria, Bacteroidota, and Firmicutes were the dominant bacterial phyla (abundance > 1%) in the rumen, with the dominant phyla in the YC group also including Spirochaetota. The relative abundance of Proteobacteria in the CK1 group was significantly higher (*p* < 0.05) than that in other groups, indicating that feeding reduced the relative abundance of Proteobacteria. The relative abundance of Spirochaetota and Fibrobacterota in the YC group was significantly higher (*p* < 0.05) than in the other three groups, and the relative abundance of Firmicutes in the XP group was significantly higher (*p* < 0.05) than that in the YC and CK1 groups (Figure 4A). *Acinetobacter*, *Succiniclasticum*, *Rikenellaceae*_RC9_gut_group, *Succinivibrio,* and *Prevotella*_7 were the dominant bacterial genera in the rumen. The relative abundance of *Acinetobacter* in the CK1 group was significantly higher (*p* < 0.05) than that in the other three groups, indicating that feeding reduced the relative abundance of *Acinetobacter*. Compared with the CK1 group, the relative abundance of *Succinivibrio* in the CK2, XP, and YC groups was significantly upregulated (*p* < 0.05), indicating that feeding significantly upregulated the relative abundance of *Succinivibrio*. Compared with the CK1 group, the relative abundance of *Dialister* was significantly increased (*p* < 0.05) in the XP and YC groups, suggesting that supplementing with yeast culture has the potential to increase the abundance of Dialister. Additionally, the relative abundance of *Rikenellaceae*_RC9_gut_group in the CK2 group was significantly higher (*p* < 0.05) than that in the YC and CK1 groups. The YC group’s relative abundance of *Ruminobacter* was significantly higher (*p* < 0.05) than that in other groups, indicating that supplementation with the self-developed yeast culture YC increased the relative abundance of *Ruminobacter* (Figure 4B).

**Figure 5:**
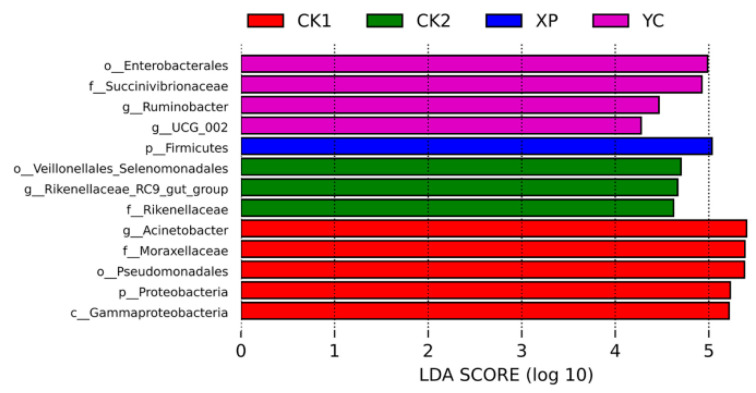
Discriminant analysis of species differences at multiple levels of LEfSe.

The results of linear discriminant analysis effect size (LEfSe) revealed a significant difference in the relative abundance of the 13 detected bacterial taxa (Figure 5). At the phylum level, the Proteobacteria in the CK1 group were significantly enriched (LDA ≥ 4, *p* < 0.05), and the Firmicutes in the XP group were significantly enriched (LDA ≥ 4, *p* < 0.05). At the genus level, the *Acinetobacter* of the CK1 group was significantly enriched (LDA ≥ 4, *p* < 0.05), the *Rikenellaceae*_RC9_gut_group of the CK2 group was significantly enriched (LDA ≥ 4, *p* < 0.05), and the *Ruminobacter* and UCG-002 of the YC group were significantly enriched (LDA ≥ 4, *p* < 0.05).

### 3.3. Rumen Fungal Diversity Analysis

**Figure 6:**
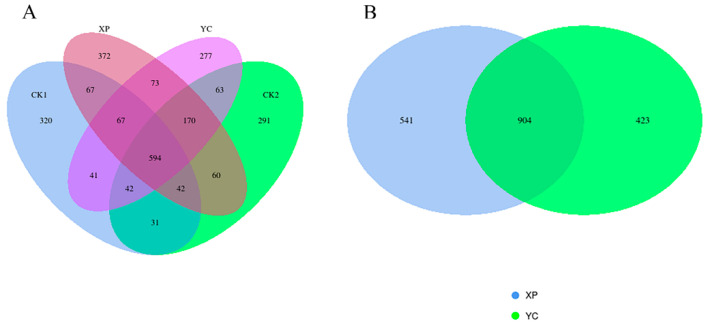
Venn diagram for all groups (**A**) and Venn diagram for trial groups (**B**) of rumen fungi.

A total of 3242 ASVs were detected in the fermentation solution of the four groups, with 1204 ASVs and 478 unique ASVs in the CK1 group, 1412 ASVs and 565 unique ASVs in the CK2 group, 1160 ASVs and 444 unique ASVs in the XP group, and 1523 ASVs and 700 unique ASVs in the YC group (Figure 6). The number of shared ASVs was 369, accounting for 11.40% of the total ASVs. The number of ASVs shared between the XP and YC groups was 561, accounting for 26.44% of the total ASVs in the trial groups.

**Figure 7:**
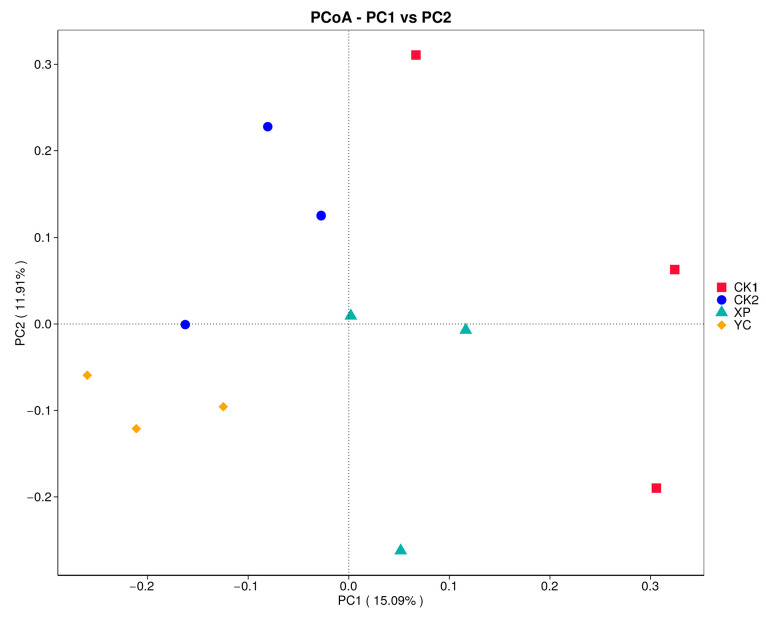
PCoA analysis of rumen fungi.

The influences of yeast culture on the alpha and beta diversity of fungal communities in the ruminal fermentation solution of Hu sheep are shown in Table 4 and Figure 7, respectively. The results showed that Good’s coverage of the four groups was all equal to 0.999, which can accurately reflect the composition of most species in the rumen of Hu sheep. Compared with other groups, the Chao1 index in the XP group was significantly reduced (*p* < 0.05). Likewise, compared with the CK2 and YC groups, the observed feature index of the CK1 and XP groups was significantly reduced (*p* < 0.05). There were no significant differences (*p* > 0.05) in the remaining diversity indexes among all groups, but the YC group tended to have higher values than those in other groups (Table 4). The PCoA results showed relative separation between groups and within groups, suggesting that adding yeast culture to the diets of Hu sheep had an impact on rumen fungal composition (Figure 7). PERMNOVA further demonstrated that there were significant differences (*p* < 0.05) in the fungi profiles among the four groups (*p* = 0.023).

Through ASV annotation and comparison, a total of eight phyla and 83 genera were detected in 12 samples from four groups. The composition of ruminal fermentation solution at the level of fungal phylum is shown in Table 5. Compared with other groups, the relative abundance of Fungi_phy_Incertae_sedis in the YC group was significantly increased (*p* < 0.05), while Basidiomycota was significantly decreased (*p* < 0.05). Furthermore, the Mucoromycota relative abundance in the XP group was significantly elevated (*p* < 0.05) when compared to the CK1 and CK2 groups. This shows that supplementing with yeast culture can increase the relative abundance of dominant fungi phyla in the rumen of Fungi_phy_Incertae_sedis and Mucoromycota, respectively.

**Figure 8:**
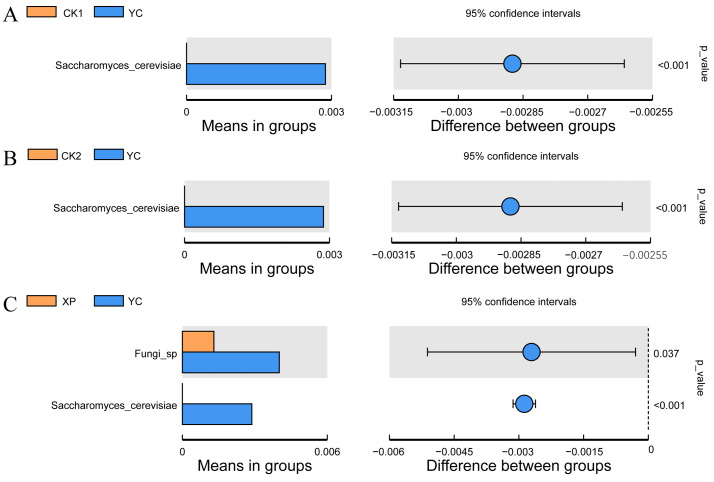
Species differences between groups. (**A**) Species with significant differences between CK1 and YC groups identified by *t*-test. (**B**) Species with significant differences between CK2 and YC groups identified by *t*-test. (**C**) Species with significant differences between XP and YC groups identified by *t*-test.

Intergroup differences in the species assessed for the fermentation solution group are shown in Figure 8. The *t*-test results showed that the relative abundance of *Saccharomyces* in the YC group was significantly higher (*p* < 0.05) than that in other groups. Compared with the XP group, the relative abundance of Fungi_phy_Incertae_sedis in the YC group was significantly increased (*p* < 0.05). Adding self-developed yeast culture can increase the relative abundance of dominant bacterial genera in the rumen.

## 4. Discussion

In the large-scale and intensive farming mode, in order to pursue high production performance, high-intensity fattening beef cattle, mutton sheep, and high-yield dairy cows are often fed with a high-concentrate diet nutrition strategy [6]. Long-term feeding of high-concentrate diets can easily lead to ruminal diseases and other health-related issues in ruminants, which bring many economic losses to producers, such as feed wastage, prolonged turnover rate, and even death [6,9]. Therefore, while considering the improvement of production performance, maintaining a relatively stable ruminal environment is a necessary prerequisite for the healthy growth and development of ruminants. Ruminal gas production, pH, ammonia nitrogen concentration, microbial protein content, and volatile fatty acid levels are important indicators for assessing ruminal fermentation in ruminant activity [28,29]. Gas production specifically reflects the fermentation activity of the diet in the rumen and the microbial metabolic activity, and it is closely related to the degradation efficiency of organic matter and metabolic energy level [28]. Studies have shown that adding different yeast cultures does not affect the gas production parameters of rumen fermentation in vitro [30]. However, the results of this study showed that from hour 4 to hour 48 after the addition of the self-developed YC and XP yeast cultures, the cumulative gas production in the fermentation solution was significantly higher than that in CK2. This indicates that the addition of the self-developed YC and XP yeast cultures significantly promoted ruminal gas production, leading to an enhancement in microbial activity within a short period of time. Moreover, the yeast culture, which is rich in proteins, polysaccharides, and other substances, promotes the growth and reproduction of ruminal microorganisms, thereby improving ruminal fermentation. In addition, some studies have shown that adding yeast culture to diet can increase rumen gas production in vitro [31]. The differences in these results may be related to experimental conditions, substrate composition, yeast culture strains, and production processes. The normal pH for the ruminal fermentation range is between 5.5 and 7.5, which is easily influenced by dietary composition [32]. In this study, the pH of the fermentation solution ranged from 6.60 to 7.03, which was within the normal ruminal fermentation range. Although high-concentrate diets were added to the experiment, pH remained relatively high and stable. This may be due to the buffer salts added to the artificial rumen nutrient solution, which helped buffer the volatile fatty acids and lactic acid produced in vitro fermentation. The effects of yeast cultures on rumen pH were also different in different studies. Studies have shown that yeast cultures can regulate rumen pH [33]. However, it has also been reported that supplementing yeast cultures in diets does not affect the pH of rumen fermentation solution in vitro [34]. In intensive breeding, buffering and maintaining optimal ruminal pH is crucial. In the two groups supplemented with yeast culture, YC significantly increased ruminal solution pH compared to XP, suggesting that YC may have a potential buffering effect on rumen.

NH_3_-N is a parameter index of nitrogen degradation in the rumen of ruminants, and it is also the raw material for microbial protein synthesis. The content of microbial protein can intuitively reflect the ability of ruminal microorganisms to utilize ammonia nitrogen [35]. In this study, adding yeast culture significantly increased microbial protein content, and the self-developed yeast culture YC showed a more pronounced effect. The increase in microbial protein content could be attributed to the action of the ruminal microbial community [36]. The addition of high-concentrate diets has increased the nitrogen-utilizing microbial community in a short period of time. Moreover, YC and XP stimulated the growth of nitrogen-utilizing microorganisms, further promoting microbial protein content. Volatile fatty acids are the main degradation products of carbohydrates by ruminal microorganisms and the primary energy source for the animal, including acetic acid, propionic acid, butyric acid, and others [37]. Acetic acid is a primary precursor for milk fat production, while propionic acid synthesizes glucose through the gluconeogenesis pathway. In ruminant production, higher propionic acid content provides more energy for animals [38]. In the CK2 group, the acetic acid content and acetic-to-propionic acid ratio were significantly increased. However, supplementation with YC and XP led to a significant decrease in the acetic-to-propionic acid ratio, indicating that supplementing yeast cultures can change the ruminal fermentation type of fattening Hu sheep from the acetic acid type to propionic acid type, thereby improving the ruminal fermentation characteristics. Zeng Yu et al. [39] also reported that dietary supplementation of yeast culture to housed yaks can reduce the acetate-to-propionate ratio and increase the propionic acid ratio, which was consistent with the results of this study. Propionic acid type fermentation is beneficial to improve the feed conversion efficiency of the animal and increase the weight gain of mutton sheep under the same feed intake [40].

In this study, we initially hypothesized that the supplementation of yeast cultures could alleviate the reduction in microbial community diversity under high-concentrate feeding conditions. However, in this study, the addition of yeast cultures did not significantly alter the alpha diversity of rumen bacteria. In this study, the dominant bacterial phyla in each group were Bacteroidota, Firmicutes, and Proteobacteria, which was consistent with previous findings in yak [41], tan sheep [42], and beef cattle [43]. The relative abundance of Proteobacteria in the fasting state accounted for more than 50%, and this phylum includes multiple pathogenic bacteria, reflecting the disorder in the ruminal microecological structure [44]. After adding the high-concentrate diets, there was a significant change in the structure of the dominant microbial community, which increased the relative abundance of bacteria-degraded various substances, such as fibers, proteins, and polysaccharides, and the ruminal microecological environment tended to be relatively stable. Compared to the XP group, the relative abundance of Proteobacteria in the fermentation solution of the YC group showed a slight increase, while the relative abundance of Firmicutes was significantly reduced. A study found that compared to lambs at high risk of SARA, those at low risk exhibited an increased relative abundance of Proteobacteria and a decreased relative abundance of Firmicutes in the rumen [45]. We predict that adding YC under the conditions of this study has the potential to prevent the risk of gastric acidosis more effectively than XP. In this study, adding YC significantly increased the relative abundance of Spirochaetota and Fibrobacterota. Spirochaetota plays an important role in decomposing plant fiber substances, such as cellulose and pectin, and converting them into volatile fatty acids [46]. Fibrobacterota is another phylum of bacteria closely related to the degradation of lignocellulose and fiber substances [39]. This indicates that adding YC to the high-concentrate diets of Hu sheep promoted the growth of fiber substances degrading bacteria and rumen microorganisms, which was beneficial for the digestion of difficult-to-degrade substances, such as cellulose, in the diet and provides energy for animals.

Petri et al. [47] found that at the genus level, *Rikenellaceae*_RC9_gut_group, *Prevotella,* and *Succinivibrio* were dominant genera in the rumen of ruminants by sequencing. Zeng Yu et al. [39] found that *Prevotella*_1, *Bacteroideales*_BS11_gut_group, and *Rikenellaceae*_RC9 were the dominant bacterial genera in rumen microbiota diversity in penned yaks. In this study, the dominant bacterial genera in the rumen of fattening Hu sheep were *Rikenellaceae*_RC9_gut_group, *Succinivibrio*, *Prevotella*, *Dialister,* and *Succiniclasticum*, which were not completely consistent with previous research results. This could be due to the rumen fluid being in contact with the air during the collection process, leading to differences in microbial composition, and it may also be caused by differences in feed composition, animal breeds, and other factors. The gut microbiota of *Rikenellaceae*_RC9_gut_group can efficiently degrade insoluble fibers and soluble polysaccharides, playing a key role in maintaining gut health [48]. In this study, the relative abundance of *Rikenellaceae*_RC9_gut_group was significantly increased after adding high-concentrate diets, while YC supplementation significantly decreased the relative abundance of *Rikenellaceae*_RC9_gut_group. There are also reports that *Rikenellaceae*_RC9_gut_group is positively correlated with acetic acid concentration and negatively correlated with propionic acid concentration [49,50]. This may also be the reason for a significant increase in the acetic acid content of the CK2 group in this study. Adding YC significantly increased the relative abundance of *Ruminobacter* and also increased the relative abundance of both *Succinivibrio* and *Prevotella*. The above three microbial communities have a high capacity to degrade various substrates in the rumen, thereby improving feed efficiency and daily weight gain in animals. Among them, Prevotella participates in various metabolic processes, such as glucose metabolism and amino acid metabolism, promoting the increase in volatile fatty acid content and having a positive impact on reducing intestinal inflammation [23,51,52,53]. LEfSe analysis revealed that there were significant differences in the relative abundance of 13 detected bacterial taxa among the four groups. Supplementation with yeast culture significantly enriched the relative abundance of functional microbial communities, such as Firmicutes and Ruminobacter. These floras can efficiently degrade various substrates in the rumen, provide nutrients and energy for the host, and improve the utilization rate of feed.

Bacteria and fungi play an important role in animal life activities and production processes, participating in physiological processes such as digestion, immune regulation, and energy metabolism [54]. Compared to bacteria, the richness and diversity of fungi in the rumen of ruminants are lower, but they play an important role in maintaining the microecological balance of the rumen [23]. Good’s coverage of all four groups was equal to 0.999, indicating that the sequencing truly represents the composition and richness of rumen fungi. In this study, the Chao1 index and observed features index were significantly reduced in the XP group, indicating that supplementation with XP yeast culture decreased the richness of rumen fungal communities under the experimental conditions. Rumen fungi play an important role in the degradation of crude fiber, producing a variety of enzymes that degrade substrates in the rumen, such as cellulases, hemicellulases, xylanases, and chitinases, which can degrade carbohydrates, proteins, and pectins into monomers [55]. Several studies have reported that Ascomycota, Basidiomycota, and Neocallimastigomycota are the dominant fungal phyla in the rumen of beef cattle [56], sheep [57], and yak [58]. In this study, the dominant fungal phyla in the rumen were Ascomycota, Fungi incertae sedis, Basidiomycota, and Mucoromycota, which is not entirely consistent with previous research findings. This discrepancy may be attributed to differences in animal breeds and feeding conditions, as well as the variations in fungal community dynamics between in vivo and in vitro studies. Ascomycota, the largest phylum among fungal microorganisms, is capable of degrading recalcitrant fibrous substances, such as cellulose and lignin [59]. The relative abundance of Ascomycota was significantly increased with the addition of high-concentrate diets, and this increase was not affected by yeast culture. In the analysis of fungal differences between groups at the genus level, the addition of YC to high-concentrate diets significantly increased the relative abundance of *Saccharomyces*. *Saccharomyces* had a positive effect on inhibiting the growth of pathogenic bacteria in rumen, enhancing the animal’s immunity, and promoting animal growth [60]. This shows that supplementing the self-developed yeast culture plays an active role in increasing the relative abundance of the beneficial fungus *Saccharomyces* in the rumen, thereby improving feed digestion.

## 5. Conclusions

In summary, in the in vitro rumen fermentation experiment with high-concentrate diets, adding 5% XP and YC yeast cultures, respectively, both can significantly promote rumen fermentation, improve the utilization efficiency of nitrogen-containing substances in feed, and change the rumen fermentation type from the acetic acid type to the propionic acid type. Further analysis showed that the addition of YC significantly increased the relative abundance of Spirochaetota and Fibrobacter at the phylum level, and these bacterial communities played an important role in degrading cellulose and other complex carbohydrates. Additionally, at the genus level, YC significantly increased the relative abundance of *Ruminobacter*, indicating that YC has a positive role in promoting the growth of fiber-degrading microbial communities in the rumen. Notably, the self-developed YC not only simplifies the production of raw materials and processes but also demonstrates a more significant effect in enhancing the relative abundance of functionally important microbial communities compared to XP, potentially offering greater advantages in practical applications. It is worth noting that the production process and dosage of yeast cultures may significantly influence their effectiveness. Therefore, future animal trials are needed to further investigate their optimal dosage and application effects under specific dietary conditions. This study not only provides a theoretical and practical foundation for optimizing the large-scale farming of Hu sheep but also validates the efficacy and mechanisms of action of the self-developed yeast culture on rumen function.

## Figures and Tables

**Table 1:** Feed ingredients and nutritional levels of the experimental mixed diet.

Ingredients, %		Nutrient Levels, %	
Corn	42.40	DM	90.48
Corn bran shotcrete	12.20	CP	9.38
Corn germ meal	5.50	Ash	11.65
Rice candy	3.60	CEE	1.82
Cottonseed meal	2.00	Ca	0.66
Chrysanthemum meal	2.00	P	0.29
Stone powder	1.80		
Baking soda	0.20		
Corn straw	29.80		
Premix	0.50		
Total	100.00		

DM, dry matter; CP, crude protein; Ash, coarse ash; CEE, crude fat.

**Table 2:** Effects of different yeast culture supplementations in diets on ruminal fermentation parameters.

Items	Groups ^1^				*p*-Value
	CK1	CK2	XP	YC	
GP, mL	19.00 ± 1.00 ^c^	45.00 ± 0.00 ^b^	96.00 ± 9.54 ^a^	103.00 ± 7.21 ^a^	<0.001
pH	7.03 ± 0.07 ^a^	6.66 ± 0.02 ^bc^	6.60 ± 0.02 ^c^	6.69 ± 0.04 ^b^	<0.001
NH_3_-N, mg/dL	27.24 ± 3.10 ^b^	33.27 ± 0.88 ^a^	35.93 ± 3.89 ^a^	33.96 ± 0.22 ^a^	0.015
MCP, mg/dL	3.71 ± 0.29 ^d^	9.61 ± 0.02 ^c^	24.79 ± 3.58 ^b^	56.05 ± 4.71 ^a^	<0.001
Total VFA, mmol/L	13.30 ± 0.70 ^b^	37.66 ± 0.74 ^a^	37.64 ± 0.57 ^a^	36.73 ± 0.03 ^a^	<0.001
Acetate, mmol/L	1.11 ± 0.17 c	13.79 ± 0.27 a	13.08 ± 0.50 b	12.63 ± 0.16 b	<0.001
Propionate, mmol/L	7.05 ± 0.34 ^b^	15.64 ± 0.43 ^a^	15.83 ± 0.51 ^a^	16.11 ± 0.17 ^a^	<0.001
Butyrate, mmol/L	2.94 ± 0.23 ^b^	4.34 ± 0.05 ^a^	4.34 ± 0.16 ^a^	4.08 ± 0.03 ^a^	<0.001
Isobutyrate, mmol/L	0.62 ± 0.04 ^c^	0.80 ± 0.03 ^b^	0.90 ± 0.08 ^a^	0.81 ± 0.01 ^b^	0.001
Valerate, mmol/L	0.95 ± 0.09 ^c^	2.08 ± 0.03 ^b^	2.30 ± 0.08 ^a^	2.09 ± 0.00 ^b^	<0.001
Isovalerate, mmol/L	0.59 ± 0.04 ^b^	0.73 ± 0.03 ^ab^	0.87 ± 0.15 ^a^	0.74 ± 0.01 ^ab^	0.016
AP	0.15 ± 0.02 ^c^	0.88 ± 0.02 ^a^	0.83 ± 0.05 ^b^	0.78 ± 0.02 ^b^	<0.001

^a~d^ Means within a row with different superscript letters are significantly different (*p* < 0.05). ^1^ Groups CK1, CK2, XP, and YC indicated that the composition of the substrate in the fermentation solution was 0.00 g high-concentrate diets, 0.40 g high-concentrate diets, 0.40 g high-concentrate diets + 5% XP yeast culture, and 0.40 g high-concentrate diets + 5% YC yeast culture, respectively. GP, cumulative gas production at 48 h; NH_3_-N, ammonia nitrogen; MCP, microbial protein; VFA, volatile fatty acid; AP = acetate/propionate.

**Table 3:** Alpha diversity index analysis of bacteria.

Items	Groups ^1^				*p*-Value
	CK1	CK2	XP	YC	
Chao1 index	699.47 ± 69.20 ^b^	792.42 ± 74.07 ^ab^	885.86 ± 98.94 ^a^	869.05 ± 68.16 ^a^	0.069
Observed feature	696.33 ± 67.84 ^b^	788.33 ± 71.93 ^ab^	882.33 ± 99.01 ^a^	863.33 ± 67.47 ^a^	0.067
Good’s coverage	1.000	1.000	1.000	1.000	0.441
Shannon index	4.88 ± 0.23 ^b^	7.21 ± 0.18 ^a^	7.45 ± 0.32 ^a^	7.35 ± 0.17 ^a^	<0.001
Simpson index	0.80 ± 0.02 ^b^	0.98 ± 0.00 ^a^	0.98 ± 0.00 ^a^	0.98 ± 0.00 ^a^	<0.001

^a,b^ Means within a row with different superscript letters are significantly different (*p* < 0.05). ^1^ Groups CK1, CK2, XP, and YC indicated that the composition of the substrate in the fermentation solution was 0.00 g high-concentrate diets, 0.40 g high-concentrate diets, 0.40 g high-concentrate diets + 5% XP yeast culture, and 0.40 g high-concentrate diets + 5% YC yeast culture, respectively.

**Table 4:** Alpha diversity index analysis of fungi.

Items	Groups ^1^				*p*-Value
	CK1	CK2	XP	YC	
Chao1 index	670.03 ± 76.94 ^a^	718.95 ± 36.08 ^a^	538.61 ± 33.86 ^b^	766.91 ± 49.98 ^a^	0.004
Observed feature	522.00 ± 70.74 ^b^	702.00 ± 30.51 ^a^	565.33 ± 84.10 ^b^	782.67 ± 89.65 ^a^	0.008
Good’s coverage	0.999	0.999	0.999	0.999	0.859
Shannon index	5.84 ± 1.09	6.43 ± 0.44	6.42 ± 0.15	6.86 ± 0.38	0.326
Simpson index	0.93 ± 0.04	0.95 ± 0.03	0.96 ± 0.00	0.97 ± 0.01	0.316

^a,b^ Means within a row with different superscript letters are significantly different (*p* < 0.05). ^1^ Groups CK1, CK2, XP, and YC indicated that the composition of the substrate in the fermentation solution was 0.00 g high-concentrate diets, 0.40 g high-concentrate diets, 0.40 g high-concentrate diets+ 5% XP yeast culture, and 0.40 g high-concentrate diets + 5% YC yeast culture, respectively.

**Table 5:** Effects of different yeast cultures on dominant phylum composition of ruminal fungi communities (%).

Items	Groups ^1^				*p*-Value
	CK1	CK2	XP	YC	
Ascomycota	2.69 ± 0.77 ^b^	4.69 ± 1.00 ^a^	3.22 ± 0.32 ^ab^	3.65 ± 0.86 ^ab^	0.068
Fungi_phy_Incertae_sedis	0.10 ± 0.08 ^c^	0.29 ± 0.09 ^b^	0.16 ± 0.05 ^bc^	0.43 ± 0.06 ^a^	0.002
Basidiomycota	0.21 ± 0.06 ^a^	0.19 ± 0.06 ^b^	0.13 ± 0.05 ^ab^	0.07 ± 0.07 ^c^	0.033
Mucoromycota	0.15 ± 0.03 ^b^	0.11 ± 0.01 ^b^	0.25 ± 0.08 ^a^	0.19 ± 0.02 ^ab^	0.027
Others	94.75 ± 2.91	95.02 ± 2.36	94.96 ± 3.04	95.71 ± 1.03	0.966

^a~c^ Means within a row with different superscript letters are significantly different (*p* < 0.05). ^1^ Groups CK1, CK2, XP, and YC indicated that the composition of the substrate in the fermentation solution was 0.00 g high-concentrate diets, 0.40 g high-concentrate diets, 0.40 g high-concentrate diets + 5% XP yeast culture, and 0.40 g high-concentrate diets + 5% YC yeast culture, respectively.

## Data Availability

The original contributions presented in this study are included in the article. Further inquiries can be directed to the corresponding authors.
